# The Devil is in the Details: Facets of Impulsivity as Correlates of Social Circle Change Over Time

**DOI:** 10.1093/geroni/igaf122.3855

**Published:** 2025-12-31

**Authors:** Sydney Demchak, Mary Cox, Michael Boudreaux, Thomas Oltmanns, Patrick Hill

**Affiliations:** Washington University in St. Louis, Pittsburgh, Pennsylvania, United States; Washington University in St. Louis, St. Louis, Missouri, United States; Hogan Assessments, Tulsa, Oklahoma, United States; Washington University in St. Louis, St. Louis, Missouri, United States; Washington University in St. Louis, Saint Louis, Missouri, United States

## Abstract

Impulsivity is a multifaceted trait that influences life outcomes such as financial stability, purposefulness, and connection. The current study examined the relationship between impulsivity and changes in social network during older adulthood. We employed data gathered over the span of three years from participants who were part of the St. Louis Personality and Aging Network. Impulsivity-related traits, such as self-discipline, deliberation, and excitement-seeking, were also assessed to determine more specific associations with the size and stability of older adults’ inner circle, middle circle, and outer circle social ties across two time points. Social circle information was collected from participants (n = 815, mean age: 62.5 years) across two waves, and the NEO Personality Inventory served as the baseline for the level of impulsivity and impulsivity-related traits. Results indicated that global impulsivity showed no significant associations with inner or middle circle size, or with overall social changes over time. However, the facet of self-discipline was positively related to the number of inner circle members at Wave 1 (r = .08) and Wave 2 (r = .07), and to middle circle members at both waves (r = .09 each). Additionally, deliberation was associated with fewer individuals dropping out of the inner circle over time (r = –.08). Excitement-seeking though showed no significant correlations. These findings suggest that viewing impulsivity as a singular variable would be insufficient, as it could obscure potentially relevant trait-level associations. Moreover, self-discipline and deliberation could prove important for maintaining and growing social networks in older adulthood.

